# Supplementary material for: Geographic Structure Without Co‐Divergence: Genomic Insights Into a Highly Specific Symbiosis Between Siphamia Cardinalfish and Their Bioluminescent Symbiont
Source: Ecol Evol. 2026 Mar 21;16(3):e73200. doi: 10.1002/ece3.73200 (PMC13093359; doi:10.1002/ece3.73200)
Supplement: Supplementary file 1 — Figure S1: ece373200‐sup‐0001‐FigureS1.docx. [file ECE3-16-e73200-s002.docx]

Supplementary Figure 1. A principal component analysis of *Siphamia tubifer, S. fuscolineata,* and *S. mossambica* based on 15,506 SNPs. Included are eigenvalues showing the best-fit PCA axis.
