# Supplementary material for: Geographic Structure Without Co‐Divergence: Genomic Insights Into a Highly Specific Symbiosis Between Siphamia Cardinalfish and Their Bioluminescent Symbiont
Source: Ecol Evol. 2026 Mar 21;16(3):e73200. doi: 10.1002/ece3.73200 (PMC13093359; doi:10.1002/ece3.73200)
Supplement: Supplementary file 2 — Figure S2: ece373200‐sup‐0002‐FigureS2.docx. [file ECE3-16-e73200-s003.docx]

Supplementary Figure 2. A pangenome analysis of *Photobacterium mandapamensis*.
